# LcmUNet: A Lightweight Network Combining CNN and MLP for Real-Time Medical Image Segmentation

**DOI:** 10.3390/bioengineering10060712

**Published:** 2023-06-12

**Authors:** Shuai Zhang, Yanmin Niu

**Affiliations:** School of Computer and Information Science, Chongqing Normal University, Chongqing 401331, China; 2021110516030@stu.cqnu.edu.cn

**Keywords:** medical image segmentation, lightweight network, UNet, CNN, MLP

## Abstract

In recent years, UNet and its improved variants have become the main methods for medical image segmentation. Although these models have achieved excellent results in segmentation accuracy, their large number of network parameters and high computational complexity make it difficult to achieve medical image segmentation in real-time therapy and diagnosis rapidly. To address this problem, we introduce a lightweight medical image segmentation network (LcmUNet) based on CNN and MLP. We designed LcmUNet’s structure in terms of model performance, parameters, and computational complexity. The first three layers are convolutional layers, and the last two layers are MLP layers. In the convolution part, we propose an LDA module that combines asymmetric convolution, depth-wise separable convolution, and an attention mechanism to reduce the number of network parameters while maintaining a strong feature-extraction capability. In the MLP part, we propose an LMLP module that helps enhance contextual information while focusing on local information and improves segmentation accuracy while maintaining high inference speed. This network also covers skip connections between the encoder and decoder at various levels. Our network achieves real-time segmentation results accurately in extensive experiments. With only 1.49 million model parameters and without pre-training, LcmUNet demonstrated impressive performance on different datasets. On the ISIC2018 dataset, it achieved an IoU of 85.19%, 92.07% recall, and 92.99% precision. On the BUSI dataset, it achieved an IoU of 63.99%, 79.96% recall, and 76.69% precision. Lastly, on the Kvasir-SEG dataset, LcmUNet achieved an IoU of 81.89%, 88.93% recall, and 91.79% precision.

## 1. Introduction

Medical image segmentation plays a crucial role in modern medical practice, assisting doctors in making accurate and timely diagnoses in complicated cases. Segmentation separates specific structures or regions, such as organs, lesions, and tumors, providing critical information for doctors for the effective medical diagnoses and treatments [1,2]. In the field of medical image segmentation, various techniques are employed within traditional computer vision methods. For instance, superpixel segmentation methods [3,4] combine pixels to create compact and similar regions, enhancing computational efficiency and providing higher-level representations for subsequent tasks. Watershed segmentation [5,6] relies on the gray-level gradient of an image to divide it into regions, simulating the flow of water on a terrain. Active contour methods [7,8] utilize curve evolution and energy minimization strategies to adjust contour positions for image segmentation. These methods possess their own advantages and limitations across different application scenarios. Superpixel segmentation methods excel at rapidly generating compact and relatively uniform segmentation results, making them suitable for efficiently processing large-scale images. Watershed segmentation performs well in handling images with clear boundaries, but it may encounter challenges when dealing with texture-rich or overlapping regions. Active contour methods are effective for precise shape segmentation, although they may require manual initialization or parameter adjustment for complex image structures.

In recent years, medical image segmentation based on deep learning has been widely used due to the continuous development of artificial intelligence and computer vision [9,10,11]. By using deep neural network models and large amounts of medical image data for training and learning, this method achieves automated segmentation and annotation tasks on medical images and features high efficiency, accuracy, and stability. Meanwhile, it is widely used across medical imaging segmentation containing CT images [12,13,14], X-ray images [15,16], MRI images [17,18,19], OCTA images [20,21], and ultrasound images [22,23,24], playing a key role in medical diagnosis, treatment, and monitoring. However, currently, medical image segmentation algorithms based on deep learning mainly face three primary problems:

- Large number of parameters and high computational cost: Medical image data typically require more computing resources and storage capacity to process and store the required data due to their high resolution, multiple channels, and complex structural features. Deep learning-based medical image segmentation networks require a significant amount of training and optimization, leading to high computational costs and a large number of parameters.
- Insufficient accuracy of lightweight segmentation models: Lightweight image segmentation models are essential for some low-power device applications. However, currently, many lightweight models perform worse than other advanced models in segmentation accuracy, especially regarding small or indistinguishable areas that are trapped with errors or omission.
- Insufficient extraction of global and local information makes distinguishing between the boundaries of the segmentation area and background challenging: Organs and lesions in medical images often have complex shapes and structures, requiring the full extraction and coordination of global and local information during segmentation. As deep learning models typically only focus on small receptive fields, it is difficult to tell apart the boundaries between the segmentation area and background, leading in the inaccurate results.

To solve these problems, we innovatively propose a lightweight network called LcmUNet, where L stands for lightweight, c for CNN, and m for mlp, and design two modules: the LDA module where D for depth-wise separable convolution and A for asymmetric convolution and the LMLP module. This network can reduce computational complexity, improve inference speed, and achieve a better balance between speed and accuracy. In addition, because pooling operations result in a significant loss of positional information, the features extracted by the encoder are insufficient to accurately segment pixels, which leads into rough segmentation images generated by the decoder through upsampling. Therefore, we have used the UNet structure to establish skip connections between the encoder and decoder so that high-resolution features can participate in subsequent decoding processes, helping the decoder to better restore the details of the target. We have validated LcmUNet on three medical image segmentation datasets and achieved excellent results.

**Figure 1:**
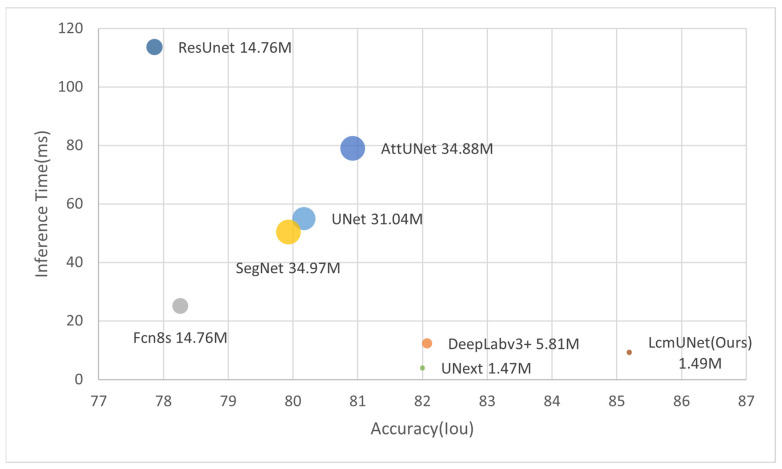
Trade-off between accuracy and efficiency compared to state-of-the-art networks (circle size represents number of parameters).

We compared LcmUNet with the current state-of-the-art methods on the ISIC2018 dataset, and it demonstrated excellent semantic segmentation accuracy and fast inference speed, as illustrated in Figure 1.

The main contributions of this paper can be summarized as follows:

- We propose a novel lightweight neural network, called LcmUNet, that significantly improves the accuracy of medical image segmentation tasks while maintaining a high inference speed.
- To address the issue of large parameter size in traditional models, we design the LDA module, which utilizes depth-wise separable convolution, asymmetric convolution, and an attention mechanism to balance the inference speed and segmentation accuracy. Introducing the LDA module in the convolution stage reduces the number of network parameters while enhancing the feature extraction capabilities.
- Additionally, we propose an MLP module called LMLP, which fuses context information and operates in different directions to further enhance information expression and improve segmentation accuracy.
- Finally, we test and demonstrate the performance of LcmUNet on three medical image segmentation datasets: ISIC2018, BUSI, and Kvasir-SEG. Specifically, with only 1.49M parameters, we obtained segmentation accuracies of 85.19%, 63.99%, and 81.89% on the three datasets using a NVIDIA 3060 GPU, further highlighting the superiority of LcmUNet in the field of medical image segmentation.

The rest of the paper is organized as follows: Section 2 discusses related works on UNet, lightweight networks, and MLP. Then, Section 3 provides a detailed description of LcmUNet’s architecture and relevant principles. Furthermore, Section 4 analyzes the experimental results. Finally, Section 5 and Section 6 summarize the contributions of LcmUNet and outlines future work.

## 2. Related Works

### 2.1. UNet

Among deep learning frameworks, UNet has established itself as one of the most popular models for medical image segmentation. Initially developed by Ronnenberger et al. for cell segmentation, its symmetric model structure reduces image size and dimensions, making it well-suited for high-dimensional medical images [25]. 

Several variations of UNet have been proposed to address common challenges in medical image segmentation. Attention UNet introduces a soft attention mechanism that optimizes the segmentation results based on lower-level features [26]. ResUNet adds a weighted attention mechanism to enhance the network’s ability to differentiate between target and background regions [27]. RAUNet uses fusion residual modules and attention mechanisms to suppress irrelevant information and highlight image features [28]. Zhou et al. leveraged an improved skip-connection technique to combine pixel-level features at different depths, improving the model accuracy and convergence speed [29].

SD-UNet is designed for devices with limited computational resources and introduces weight normalization and group normalization methods to address performance degradation caused by deep convolutional layers [30]. DCUNet employs Res-Path connections and dual-channel modules to provide more effective feature information while using fewer parameters [31]. 

Finally, Valanarasu et al. introduced UNext, a segmentation network based on convolutional multilayer perceptrons that significantly improves segmentation results while maintaining a low computational cost, offering a new solution for medical image analysis tasks [32]. In Table 1, we provide a comprehensive summary of the strengths and weaknesses of the aforementioned networks.

### 2.2. Lightweight Models

The primary objective of lightweight networks is to overcome traditional neural network limitations in storage space and energy consumption. This is accomplished through manual design, neural architecture search, or automated machine learning techniques for reducing the storage space requirements and improving the running speed. MobileNet is based on the idea of using depth-wise separable convolutions rather than regular convolutions for feature extraction and point convolutions to combine features instead of filters. This significantly reduces the number of parameters and computations [33]. MobileNet V2 is an upgraded version that incorporates residual thinking from the ResNet network; it combines high-dimensional features with ReLU activation layers to maintain low-dimensional input information as much as possible [34,35]. 

ShuffleNet uses channel transformation to randomly and uniformly disrupt the feature maps obtained after group convolution on the channels, which ensures that the different groups of the input are in the group convolution operation [36]. The 1 × 1 convolution in the ShuffleNet V2 basic unit no longer uses group convolution, but regular convolution, compensating for the overuse of group convolution [37]. Xception, a lightweight network structure designed by Google based on spatial and channel correlations, builds upon Inception V3 [38]. Unlike depth-wise separable convolution’s approach of performing spatial correlations followed by channel correlations, Xception first obtains channel correlations before learning spatial correlations. 

GhostNet uses linear transformations to obtain Ghost features, which enables more feature mappings at a lower cost [39]; RepGhost develops a hardware-efficient RepGhost module based on structural reparameterization technology, achieving the implicit reuse of features [40]. A lightweight model can be achieved through lightweight methods based on neural network structure search and automatic model compression, such as MnasNet and model compression [41]. In Table 2, we provide a comprehensive summary of the strengths and weaknesses of the aforementioned networks.

These lightweight networks can operate on hardware-limited devices while maintaining high classification accuracy and segmentation accuracy, making them of immense practical value.

### 2.3. MLP

The pure MLP structure is an effective technique for image segmentation that offers advantages such as fast inference speed and simple, efficient structures. Image segmentation models based on Transformer [42] do not require heavy decoders due to their large receptive field range, making it possible to yield excellent results using simple MLP structures alone. To enhance image segmentation, new pure MLP structures have been proposed.

Reparameterization MLP (RepMLP) incorporates local prior information for image recognition through a reparameterization technique [43]. By processing input features during training with convolutional layers, it achieves powerful image recognition by merging them with fully connected layers during inference. Residual MLP (ResMLP), on the other hand, employs two residual operations to update features and uses flattened image blocks as inputs. It accomplishes classification without any attention mechanism, only through linear layers and GELU nonlinear activation functions [44]. External Attention MLP (EAMLP) includes a multi-head external attention mechanism (achieved with two linear layers) within an MLP network structure [45]. Cycle-FC, as proposed in CycleMLP, aligns features from different tokens to the same channel before reducing network computations via channel mapping. This approach remains insensitive to image resolution [46]. ConvMLP uses a convolutional tokenizer to produce initial feature maps, improving spatial connections while reducing computation [47].

Overall, the pure MLP structure provides unique advantages in image processing. In recent years, numerous types of MLP network structures have emerged, opening up more efficient and convenient methods and ideas in related fields. Considering these advancements, MLP-based image segmentation models will continue to hold promise in research applications.

In Table 3, we provide a comprehensive summary of the strengths and weaknesses of the aforementioned networks.

## 3. LcmUNet

To reduce the model’s parameter count without compromising on the accuracy, we have introduced a novel lightweight medical image segmentation network called LcmUNet. Our network combines CNN and MLP and consists of two stages: convolution and MLP.

In the convolution stage, we use three LDA modules to downsample the input and maintain good feature-extraction ability while reducing model complexity. In the MLP stage, we utilize two layers of LMLP modules that fuse contextual information for deep feature extraction. These modules emphasize high-level semantic information, guiding the model to pay attention to local information and enhancing its feature representation ability.

**Figure 2:**
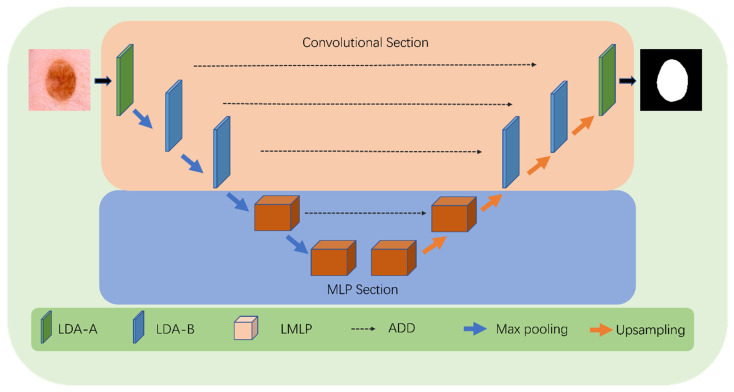
Detailed structure of LcmUNet.

The above describes the design of the encoder. For the decoder, we adopt a symmetric design to the encoder. To better integrate low-level detailed features and high-level semantic features in the encoder, we use the UNet architecture throughout the entire model. The parts of the encoder and decoder are connected by skip connections, as shown in Figure 2.

In the following section, we focus on the details of each module to gain a deep understanding of the advantages and performance of our proposed method.

### 3.1. LDA Module

**Figure 3:**
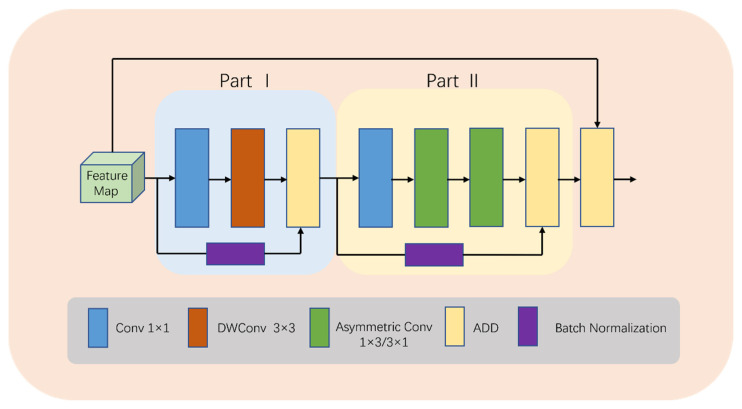
Detailed structure of LDA-A.

To balance model complexity and accuracy in medical image segmentation, our novel lightweight module called LDA-A (shown in Figure 3) is designed. In the first stage, conventional convolution uses downsampling to capture features, followed by depth-wise separable convolution to reduce computation by mapping feature channels. Additionally, BN operations add more nonlinear features, making the model more efficient during the training process while integrating feature map information quickly through ADD operations.

In the second stage, asymmetric convolution replaces depth-wise separable convolution to reduce the computational complexity and redundancy. Our design considers multiple factors: Firstly, due to the current low number of channels, asymmetric convolution is more efficient. Secondly, the addition of this operation expands the feature space, allowing the model to adapt to targets with different aspect ratios, ultimately resulting in better learning outcomes. Finally, residual connections are utilized to construct a high-performance deep network that avoids gradient vanishing or explosion problems, while ADD operations fuse shallow and deep information.

It is important to note that in the entire LDA module, ADD operation is used for feature fusion, avoiding Concat operation due to its complex memory copies on hardware devices, consequently causing significant computational costs. Although Concat operation is parameter-free and Flops-free, our experience suggests that utilizing ADD operation for feature fusion effectively reduces the computational cost in practice.

The computation in the LDA-A block can be summarized as:

Y_PO1_ = Conv_1_(X), (1)

Y_PO2_ = DWConv(Y_PO1_), (2)

Y_PO3_ = ADD(Y_PO2_,BN(Conv(X))), (3)

Y_PT1_ = Conv_1_(Y_PO3_), (4)

Y_PT2_ = ASConv_1×3_(Y_PT1_), (5)

Y_PT3_ = ASConv_3×1_(Y_PT2_), (6)

Y_PT4_ = ADD(Y_PT3_,BN(Conv(Y_PO3_))), (7)

Y = ADD(Y_PT4_,X), (8)

where X denotes features, PO denotes Part I, PT denotes Part II, DWConv denotes depth-wise convolution, Conv_1_ denotes conv 1 × 1, ASConv denotes asymmetric convolution, and BN denotes batch normalization.

**Figure 4:**
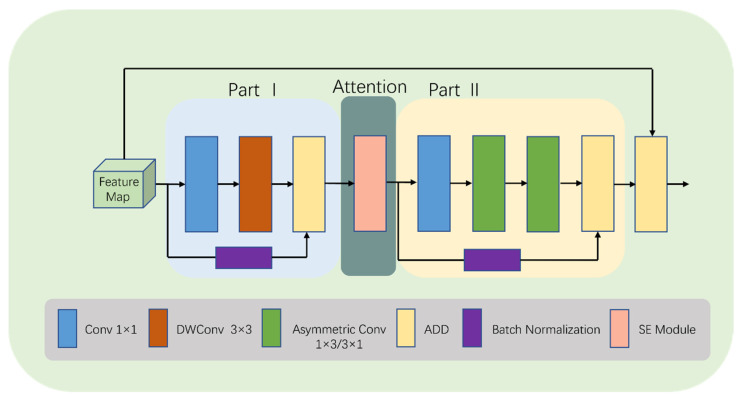
Detailed structure of LDA-B.

To increase the model’s interest in the target region while giving greater weight to effective feature maps and reducing the weight of ineffective or less effective ones, we make some changes in the last two modules of the convolution stage. This new module is called LDA-B (as shown in Figure 4). 

**Figure 5:**
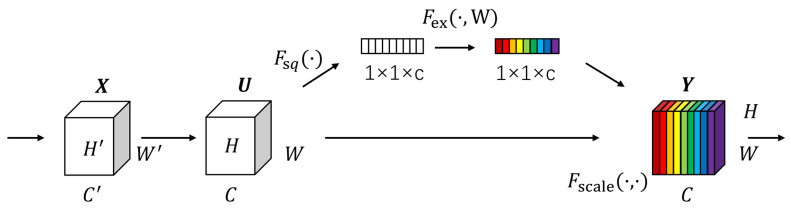
Detailed structure of SE module.

Specifically, we add an SE [48] module after the first ADD operation in LDA (as shown in Figure 5). The SE module selectively enhances channel performance based on global information and suppresses channels that perform poorly. Although this inevitably increases the number of parameters and computations, learning channel correlations and filtering attention for specific channels brings performance improvements to the model with minimal additional computational costs. It is worth noting that using the SE module not only enhances the focus on the target area, but also improves the model’s generalization ability.

The SE module mainly consists of two operations: squeeze and excitation. The output of the squeeze operation is used as the input of the excitation operation, and the output of the excitation operation is multiplied with the original input features to obtain the final output of the SE module. The squeeze operation can be expressed as:

(9) $$Z=F_{\mathrm{sq}}(f)=\frac{1}{H\times W}\sum_{i=1}^{H}\cdot \sum_{j=1}^{w}\left(i,j\right)$$

where Z is the output of the squeeze operation; F_sq_ is the squeeze operation function; f ∈ R^H × W^ is the two-dimensional feature map set; f (i,j) is one of the elements; and H and W denote the height and width of the feature map space information, respectively.

The excitation operation can be expressed as:

(10) $$S=F_{\mathrm{ex}}(Z,\;W)=\sigma (W_{2}\delta (W_{1}Z))$$

where S is the output of the excitation operation; F_ex_ is the excitation operation function; σ and δ denote the Sigmoid and ReLU excitation functions, respectively; W_1_ ∈ R^(C/r) × C^ and W_2_ ∈ R^C × (C/r)^, where W_1_ and W_2_ are some elements, respectively; and r is the reduced dimensional coefficient. 

After the excitation operation, the resulting output weights are multiplied with the original input features to have:

y = F_scale_(f, S) = S⋯f(i, j) (11)

where F_scale_ is the scale operation; y is a value in the final output of the SE module, y = [y_1_, y_2_, …, y_c_].

To represent the equations of LDA-B, it is appropriate to include Equations (9)–(11) immediately following Equation (3).

### 3.2. LMLP Module

When performing semantic segmentation tasks, the boundaries between the segmented area and the background are often unclear or blurred. Existing approaches aim to improve the segmentation performance by incorporating more local information. However, these methods often have high computational complexity and numbers of parameters, limiting their application in lightweight module scenarios.

**Figure 6:**
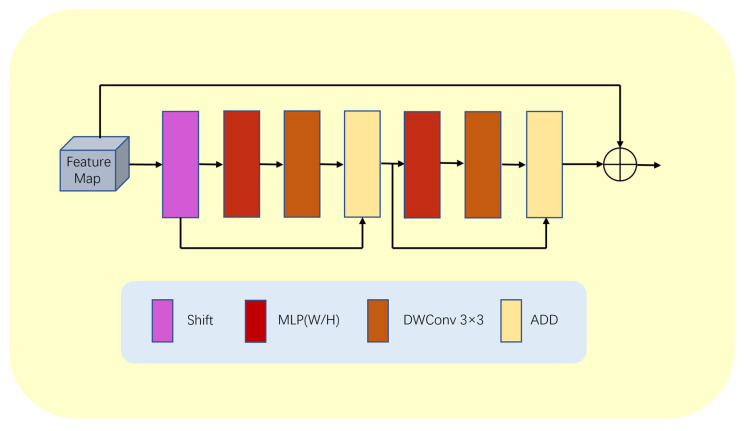
Detailed structure of LMLP module.

To solve this problem, this paper proposes a lightweight multilayer perceptron (LMLP) module, as illustrated in Figure 6. This module first performs a shift operation on the input image to convert features from a one-dimensional sequence to a token sequence. Then, it applies MLP operations on the token sequence in the width direction. Next, the module passes the features through a deep convolutional layer to reduce the number of parameters while enhancing the positional information and correlations between features. The tokens then undergo MLP operations on the height dimension, followed by another deep convolutional layer for further feature extraction. Since MLP operations are performed at the deep level of the model and the input features are relatively abstract and complex, performing MLP operations in both the height and width directions allows us to better focus on local information and effectively tackle the blurring problem at the junction of the background and segmented areas.

Furthermore, our LMLP module utilizes two ADD operations to integrate more feature information. The first ADD operation adds the original token to the features passed on from the deep convolutional layer, while the second ADD operation combines the features before MLP in the height direction with the features processed by the deep convolutional layer. These multiple ADD operations enhance the module’s feature expression ability, making the model more adaptable and robust.

Finally, we utilize layer normalization (LN) at the output end of the LMLP module to eliminate the mean offset and improve the training stability. This ensures that the module produces stable output results, which are then passed on to the next block to complete the entire segmentation task.

Furthermore, we add a residual connection inside the LMLP module to fuse feature maps of different resolutions. This component has two branches, one responsible for high-resolution segmentation tasks and one for low-resolution global semantic feature learning tasks. This multi-branch structure enables our LMLP module to effectively handle complex image structures and objects.

The computation in the LMLP block can be summarized as:

X_T_ = Shift(Tokenize(X)), (12)

Y_W1_ = MLP_W_(X_T_), (13)

Y_W2_ = DWConv(Y_W1_), (14)

Y_W3_ = ADD(Y_W2_ + X_T_), (15)

Y_H1_ = MLP_H_(Y_W3_), (16)

Y_H2_ = DWConv(Y_H1_), (17)

Y_H3_ = ADD(Y_W3_ + Y_H2_), (18)

Y = X + Y_H3_, (19)

where X denotes the features, W denotes an operation in the width direction, H denotes an operation in the height direction, and DWConv denotes a depth convolution.

In summary, our LMLP module is a lightweight multilayer perceptron designed to address semantic segmentation problems. It possesses good locality, a multi-branch structure, and advantages in computational and spatial efficiency.

### 3.3. LcmUNet Architecture

Our LcmUNet adopts a lightweight encoder–decoder architecture, as outlined in Table 4. The model begins with the LDA-A module as the first layer. In the first stage, conventional convolution is applied for downsampling and feature acquisition. Following that, depth-separable convolution is utilized to reduce computational requirements while mapping the feature channels. In the second stage of our architecture, the depth-separable convolution is replaced with asymmetric convolution to expand the feature space, enabling the model to effectively handle targets with diverse aspect ratios. Moving on to the second to third layers, we have the LDA-B module, which incorporates the SE (squeeze and excitation) module between the first and second stages. This addition enhances the focus on the target region, distinguishing it from the LDA-A module. 

Continuing with the architecture, the fourth to fifth layers consist of LMLP modules. These modules operate in different directions, allowing for comprehensive feature extraction. At the end of each MLP, a depth-separable convolution is incorporated to reduce the number of parameters while simultaneously enhancing the location information of the feature representation and the correlation between features.

To ensure efficient computational performance, we considered the computational overhead, and thus, the maximum number of channels in our model is limited to 256. This limitation helps maintain a balance between computational efficiency and model performance.

## 4. Experiment

We conducted segmentation experiments on three datasets, ISIC2018, BUSI, and Kvasir-SEG, to demonstrate the low parameter count and high segmentation accuracy of our proposed LcmUNet. In addition, we performed ablation studies to gain a better understanding of the potential behavior of semantic segmentation networks in computer vision.

### 4.1. Implement Details

To ensure the fairness of experimental data, all comparison networks in this study were trained using NVIDIA RTX 3060 12GB GPU. We evaluated the performance of LcmUNet on three different datasets: ISIC2018, BUSI, and Kvasir-SEG. 

The ISIC 2018 dataset, obtained from the ISIC 2018 Challenge, comprises 2574 images divided into seven different categories. Each image has a size of 650 × 450 pixels. The dataset can be accessed at https://challenge.isic-archive.com/landing/2018/ (accessed on 25 March 2023).The breast ultrasound dataset, sourced from the Kaggle Challenge, includes normal, benign, and malignant examples. It consists of 780 ultrasound images obtained from 600 female patients aged between 25 and 75 years. The average image size in this dataset is 500 × 500 pixels. This dataset can be found at https://www.kaggle.com/datasets/aryashah2k/breast-ultrasound-images-dataset/code (accessed on 25 March 2023). The Kvasir-SEG dataset is an open-access dataset containing gastrointestinal polyp images and their corresponding segmentation masks. These masks have been manually annotated and validated by experienced gastroenterologists. The dataset comprises 1000 polyp images from the Kvasir dataset v2, with image resolutions ranging from 332 × 487 to 1920 × 1072 pixels. The dataset can be accessed at https://datasets.simula.no/kvasir-seg/ (accessed on 25 March 2023).

In this study, all images were resized to 256 × 256 pixels, and 80% of the samples were used for training and validation, while the remaining 20% were used for testing. LcmUNet was trained end-to-end on the aforementioned datasets using the Adam optimizer. To ensure that the model was fully trained, we set 100 epochs for training, with a batch size of eight chosen to utilize the GPU memory optimally. Notably, the initial learning rate was set to 1 × 10^−3^, the power of the learning rate was 0.9, and the weight decay was set to 5 × 10^−4^.

Furthermore, medical image segmentation often suffers from data scarcity, which may lead to overfitting. Therefore, in this paper, we performed data augmentation through angle rotation, random scale zooming, contrast and brightness adjustment, as well as cropping to enhance the model’s generalization and improve its robustness.

To obtain better experimental results, we trained the model with a combination of binary cross-entropy loss as well as dice loss. The loss between the prediction y’ and the target y is formulated as:

Loss = Dice (y’, y) + 0.5 BCE (y’, y),(20)

### 4.2. Comparative Experiments

To demonstrate the superiority of our network, we compared our approach to seven state-of-the-art models including Fcn8s [49], SegNet [50], UNet, DeepLabv3+ [51], AttUNet, ResUnet, and UNext. The experimental results of these models were obtained using the default parameter settings provided by their respective authors. The performance evaluations and comparisons of all networks were based on widely used semantic segmentation evaluation measures: Intersection over union (Iou), recall, precision, and F1-score. Iou represents the ratio of intersection to union between the predicted and true values for each class and is calculated as:

Iou = TP/(FN + FP + TP),(21)

The recall is the ratio of the number of samples correctly predicted by the model to be in the positive category to the number of samples in all actual positive categories. As one of the classification model evaluation metrics used to measure the ability of the model to correctly identify samples in the positive category, the recall is calculated as follows:

recall = TP/(TP + FN),(22)

The precision measures how many of the samples predicted by the model to be in the positive category are actually in the positive category. It is the ratio of the number of samples correctly predicted to be in the positive category to the number of all samples predicted to be in the positive category. The precision is calculated as follows:

precision = TP/(TP + FP),(23)

The F1-score is another commonly used similarity measure to evaluate the agreement between two samples, taking values from 0 to 1, with higher values indicating better similarity. The optimal segmentation result has a value of 1, while the worst has a value of 0. The F1-score is calculated as follows:

F1-score = 2TP/(FN + FP + 2TP),(24)

where TP, FN, and FP represent true positives, false negatives, and false positives, respectively.

### 4.3. Model Complexity Analysis

In Table 5, we present a comparison of the complexity of our proposed LcmUNet to that of other state-of-the-art network models in medical image segmentation. Our experimental results demonstrate that LcmUNet outperforms these models in terms of low model parameter count, fast inference speed, and high accuracy. Notably, our proposed LcmUNet contains only 1.49 M network parameters and performs 0.49 G computations without ImageNet pre-training. Moreover, it achieves an Iou of 85.19% with a remarkable inference time of only 9.27 ms.

It is worth noting that the inference time of LcmUNet is slightly longer than that of UNext, but LcmUNET has a 3.19% higher Iou than UNext. Our proposed LcmUNet also outperforms other networks in terms of model complexity due to its lightweight attention mechanism without any complex operations. Moreover, it achieves real-time segmentation with an impressive inference time of 9.27 ms. The results of our experiments demonstrate the effectiveness of our proposed approach, striking a balance between higher accuracy and faster inference speed. Indeed, the superior performance of LcmUNet proves the advanced nature of our work.

**Figure 7:**
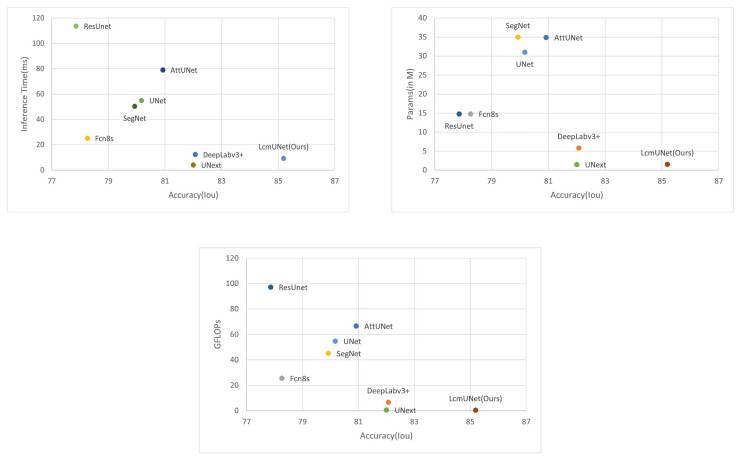
Comparison plot. The X-axis corresponds to the Iou (higher is better). The Y-axis corresponds to the inference time, number of parameters, and GFLOPs (lower is better), respectively. It can be seen that LcmUNet is the most efficient network compared to other networks.

In Figure 7, we plot the comparison charts of Iou vs. inference time, Iou vs. number of parameters, and Iou vs. GLOPs. The Iou used here corresponds to the ISIC 2018 dataset. It can be clearly seen from the charts that LcmUNet achieved outstanding performance results; in practical medical work, considering its inference time, number of parameters, and GLOPs, the study of LcmUNet is valuable.

### 4.4. Model Performance Comparison Analysis

We evaluated the performance of LcmUNet on three datasets, and Table 6 presents our experimental results, showing that our network achieved excellent segmentation results. To verify the segmentation performance of LcmUNet, we compared it with other models such as UNet, AttUNet, Deeplabv3+, ResUNet, and UNext on the same datasets. Table 1 shows the comparison between LcmUNet and the other models on the three datasets. The Iou percentages of LcmUNet were 85.19%, 81.89%, and 63.99%, respectively, while the F1-scores of LcmUNet were 91.81%, 89.92%, and 77.37%, respectively, indicating that the segmentation performance of LcmUNet was superior to the seven compared models. Overall, LcmUNet has demonstrated excellent prediction results, and its ability to predict edge information is better than other compared models, showcasing its strong robustness.

**Figure 8:**
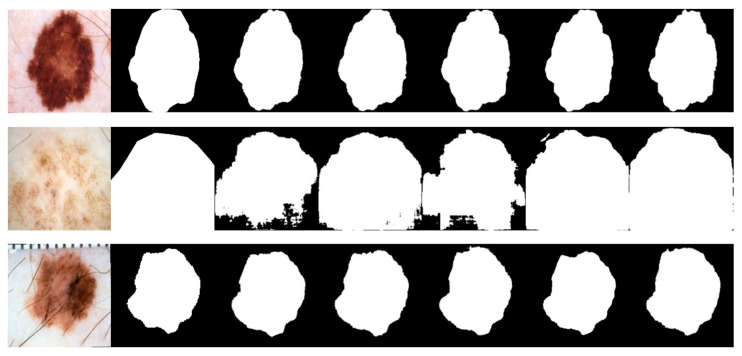
Visual detail results on the ISIC2018 dataset. From left to right are the segmentation output of the input image, GT, UNet, AttUNet, ResUNet, UNext, and our LcmUNet, respectively.

Due to the various complex scenarios such as color, texture, shape, and size variations of skin lesions; blurred boundaries; hair occlusions in the background; and interferences from surrounding tissues with similar colors, the other networks exhibited varying degrees of missed segmentation and mis-segmentation phenomena. Figure 8 displays the visual segmentation results of different methods.In contrast, LcmUNet effectively avoided complex background interference and accurately located the boundaries of the lesion area, achieving the best results in segmenting lesion areas at different scales.

**Figure 9:**
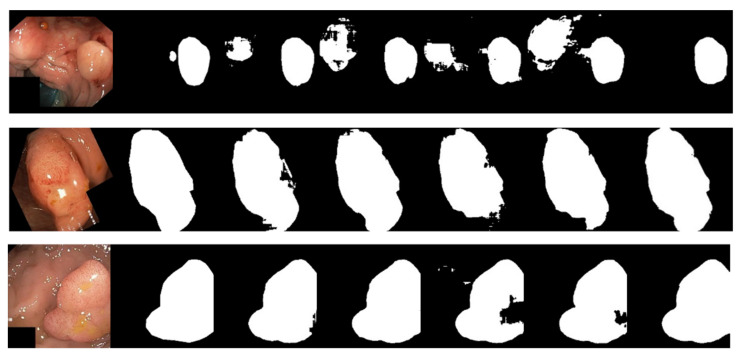
Visual detail results on the Kvasir-SEG dataset. From left to right are the segmentation output of the input image, GT, UNet, AttUNet, ResUNet, UNext, and our LcmUNet, respectively.

For the visual and intuitive comparative analysis of polyp segmentation, Figure 9 displays the visual segmentation results of different methods. It is evident in Figure 9 that colonoscopy polyps exhibit complex textures, low contrast with the surrounding mucosal tissues, and unclear boundaries, which increase the difficulty of segmentation. Other networks exhibit varying degrees of under-segmentation in some scenarios, as shown in some partially segmented results in the first row of Figure 9. Furthermore, due to the influence of uneven image brightness and low contrast, some networks have mis-segmentation areas, as shown in the third row of Figure 9. However, the experimental results demonstrate that the segmentation accuracy of LcmUNet, proposed in this paper, is higher than that of other networks and closer to the guidelines for manually annotated ground-truth images.

**Figure 10:**
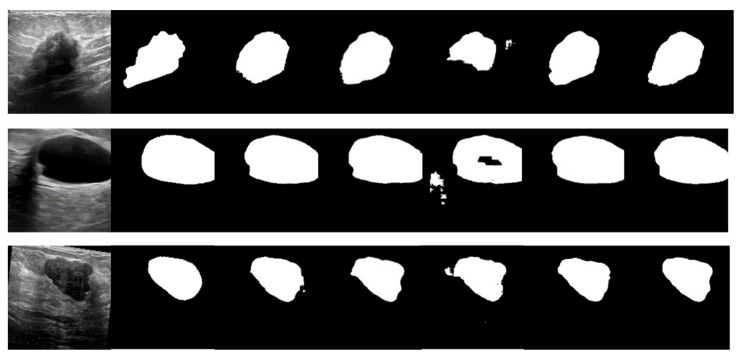
Visual detail results on the BUSI dataset. From left to right are the segmentation output of the input image, GT, UNet, AttUNet, ResUNet, UNext, and our LcmUNet, respectively.

The BUSI dataset’s visualization in Figure 10 illustrates the differences between various segmentation methods used in breast cancer tissue slice images. These images have large variations in scale, perspective, and rotation, causing differences between different images of the same case. Moreover, breast cancer and normal tissues have similarities in their morphology, color, and other aspects, increasing the difficulty of segmentation and thereby resulting in some partial segmentation results, as seen in the first row of Figure 10. The LcmUNet network, however, can focus on details and texture information, effectively improving discrimination and accurately segmenting the boundaries of lesion areas, thereby achieving the best results among many algorithms.

### 4.5. Ablation Experiments

#### 4.5.1. Overall Validation

To demonstrate the effectiveness of the two proposed modules and related designs, we conducted ablation experiments on the ISIC2018 dataset to show the role of each module, as presented in Table 7. Firstly, all modules were replaced with lightweight LDA-A modules, starting from the original UNet, to reduce the number of parameters and the complexity. It is evident that the number of model parameters decreased from 31.04 M to 0.51 M, and the accuracy increased from 80.17% to 81.69%. Secondly, except for the first-layer module, all other modules were replaced with LDA-B modules, which includes the SE attention module. The results indicate that the number of model parameters increased from 0.51 M to 0.62 M, but the accuracy of the model improved significantly from 81.69% to 83.30%. It is worth noting that increasing the parameter count by a small number results in improved model performance. Thirdly, we replaced the fourth and fifth layers of the model with LMLP modules, while the second and third layers were replaced with LDA-A modules, significantly improving the performance compared to the network consisting of five LDA-A modules. Lastly, LcmUNet was designed, consisting of the first-layer LDA-A module, second- and third-layer LDA-B modules, and fourth- and fifth-layer LMLP modules, achieving the best results in terms of accuracy and complexity.

#### 4.5.2. Number of Channels

We also addressed whether to add attention to the first-layer module, LDA-A, in the model. Typically, the number of channels in the first layer of the network is not very high. In LcmUNet, the output channel in the first layer is set at 16, making it challenging for the network to learn richer features such as texture features with different directions and frequencies. 

Therefore, we adjusted the number of channels in the SE module to study the trade-off between performance and computational cost mediated by this hyperparameter. We set the number of channels adjusted in the attention mechanism to 0 (no SE module is applied), 1/4 of the original, and 1/2 of the original. The results(as presented in Table 8) indicate that the performance under reduced channels within a certain range is robust, with better performance when there is no SE module in the first layer. This shows that the SE attention mechanism only considers attention in the channel dimension and cannot capture attention in the spatial dimension, making it more applicable to scenarios with a large number of channels.

#### 4.5.3. Number of Feature Fusions

To integrate more features, multiple ADD operations were designed in the MLP stage. The first operation is an ADD operation between the original labels and the features after the first depth-wise separable convolution, while the second operation is an ADD operation between the features before the MLP stage and the features after the second depth-wise separable convolution. Through the multiple ADD units, this layer cross-combines features in different dimensions to obtain more nonlinear and combined feature information, thereby enhancing the model’s representation capabilities. We conducted ablation experiments on the remaining two ADD operations, including two settings without ADD, one setting without the second and with the first ADD or vice versa, and both settings with ADD. The experimental results (as presented in Table 9) demonstrate that more ADD operations allow the model to obtain more feature information through combination, achieving the best performance when both ADD operations are set.

## 5. Discussion

### 5.1. A Novel Deep Learning Framework for Real-Time Medical Image Segmentation

In clinical practice, accurate segmentation of medical images is crucial for doctors to make accurate diagnoses. While deep learning has made medical image segmentation more accessible, existing methods often struggle to achieve both real-time performance and high accuracy. In order to address this challenge, we propose a novel lightweight network that combines CNN and MLP to tackle this problem.

Our proposed network leverages a lightweight design, incorporating depth-separable convolution and asymmetric convolution. This design choice ensures real-time performance during segmentation tasks. Furthermore, we introduce MLP units with a lightweight design, allowing them to operate in different directions. This enables the model to capture more local information and mitigate the negative impact of MLP, which may overlook local details.

The effectiveness of our design was confirmed through qualitative results and ablation studies. Additionally, experimental evaluations on three medical image datasets demonstrated the model’s efficacy in accurately segmenting lesion regions. Overall, our proposed lightweight network offers a promising solution to the challenge of real-time and accurate medical image segmentation, as supported by both qualitative and quantitative findings from various experiments and evaluations.

### 5.2. Limitations

While our study has yielded promising results, there are several limitations that should be acknowledged. 

Firstly, our dataset comprises 2D images, which means the network design is specifically tailored for 2D data. However, as medical datasets increasingly include 3D images, it is necessary to validate whether our model can achieve satisfactory results with 3D data using different datasets. Secondly, data augmentation techniques were employed to address the challenge of limited medical images. However, it is essential to consider the potential introduction of noise or inappropriate transformations during the augmentation process. This could affect the interpretability of the generated data and the training of the model. Therefore, exploring more appropriate methods for data augmentation is crucial. Thirdly, while we have successfully trained high-performance models and achieved encouraging results during testing, it is important to note that we have not yet ported the model to mobile devices. The limitations in this area could restrict the application scope and the effectiveness of practical implementation. However, porting the model to mobile devices is a complex task with numerous challenges and technical difficulties. For instance, mobile devices have limited computational resources and storage capacity, necessitating streamlined and optimized models to ensure efficient performance and a small storage footprint. Additionally, mobile devices operate on different operating systems and software environments compared to traditional desktop or server environments, requiring adaptation and optimization to ensure the smooth execution of models on mobile devices.

### 5.3. Future Work

Extending the algorithm to process 3D images can indeed introduce higher computational complexity. It is crucial to ensure that the algorithms maintain good performance even when dealing with large 3D data. The optimization of existing segmentation algorithms becomes essential in this context. This can involve introducing more efficient algorithms and leveraging parallel computing and hardware acceleration techniques to enhance computational efficiency.

Regarding data enhancement, it is important to avoid introducing excessive noise that may compromise the reliability of the generated images. One potential approach for future work is the use of diffusion models for data generation. The aim would be to generate images that retain the essential features and structure of real images, ensuring their reliability for medical experts. This may require the design of more complex generative models or the utilization of specific loss functions to ensure the quality of the generated data.

Lastly, an important direction for future work is to quantify and optimize the model parameters for deployment on embedded devices. This involves adapting the model to run efficiently on devices with limited computational resources. Streamlining the model, leveraging hardware acceleration, and exploring optimization techniques will be necessary to enable the deployment of the model on embedded devices.

## 6. Conclusions

Our paper proposes LcmUNet, a lightweight network that combines CNN and MLP for real-time medical image segmentation. The LDA module uses depth-wise separable convolution, asymmetric convolution, residual connection, and an attention mechanism to improve feature extraction. Meanwhile, the LMLP module leverages fusion context features with MLP operations to highlight local information in different directions. Bottom-up and top-down information are subsequently combined through skip connections. Finally, we restore the spatial information lost by the encoder due to feature map size reduction, ultimately achieving more precise boundary segmentation.

We conducted experiments on popular datasets to evaluate our approach, and the results demonstrate that LcmUNet achieves good performance in terms of segmentation accuracy and efficiency across three different medical image datasets. This work not only provides valuable references for medical images, but also proves that this model can be easily applied to other organs in medical fields. Moving forward, we aim to further optimize our model to a reduced number of parameters and deploy it on embedded devices.

## Figures and Tables

**Table 1:** Advantages and disadvantages of UNet-related networks.

Networks	Advantages	Disadvantages
Unet	Data enhancement with elastic deformation; large number of feature channels in upsampling	Excessive downsampling leads to more loss of spatial information
AttUNet	Introduces an attention mechanism that allows the network to adaptively focus on important regions of the image	Requires multiple layers of overlay to extract long-range information; relatively low efficiency
ResUNet	Adds weighted attention mechanism, enabling the model to learn more knowledge to distinguish features of target and background pixels	Large model parameters and high computational costs
RAUNet	Effectively fuses multi-level features and improves feature representation; introduces hybrid loss function to solve the class imbalance problem	Applicability may be limited by task specificity
UNet++	Enhances context-awareness by using multi-scale features; ability to retain detailed information by cascading multiscale features	Fails to express enough information in multiple scales; consumes too much memory and is difficult to train
SDUNet	Optimized for platforms with limited computational resources to reduce computational complexity and memory consumption	To accommodate platforms with low computing budgets, some performance may be lost
DCUNet	Efficient convolutional neural network with two channels is introduced, while exploiting multiple scales and contextual information	Computational complexity will increase, requiring more computational resources and time for training
UNext	Faster inference with fewer parameters and computational complexity	Weak feature-extraction ability

**Table 2:** Advantages and disadvantages of lightweight networks.

Network	Advantages	Disadvantages
MobileNet	Introduces deeply separable convolution for the lightweight design of network structures	Single network structure and overuse of activation functions lead to deactivation of neurons
MobileNetV2	Introduces the inverse residual module	Due to the small convolution kernel in the depth-separable convolution, it can easily be 0 after activation
ShuffleNet	Proposes the operation of channel shuffling to reduce the amount of computation and number of parameters by rearranging the input channels	Different numbers of input and output features, excessive use of group convolution, network fragmentation, too many element-level operations
ShuffleNet V2	Introduces channel separation, equal number of input and output features, and equal number of channels in the base unit	Running speed and storage space need to be further improved
Xception	Introduces larger convolution kernels and multi-scale feature extraction	Needs to be tuned for the task
GhostNet	Generates more features by using fewer parameters	Models are limited in their ability to represent features on complex tasks
RepGhost	Efficient implementation in hardware through reparameterization	Limited applicability on other hardware platforms

**Table 3:** Advantages and disadvantages of MLP-related networks.

Networks	Advantages	Disadvantages
RepMLP	Reparametrizes convolution operations into fully connected layers for efficient matrix operations	More sensitive to the size of the input image
ResMLP	Data efficient training strategy is adopted; translation isotropy is introduced; the idea of residual linkage is adopted	The training data requirements are relatively high
EAMLP	Uses two linear layers instead of the self-attentive mechanism to improve computational efficiency	External attention mechanisms limit the ability of the model to interact with global information between different locations
CycleMLP	Uses basic multi-layer perceptron structure and loop mechanism; performs intensive prediction tasks with limited computational resources	Limited in the ability to extract high-level features
ConvMLP	Layered architecture that enables multi-level feature extraction and representation learning of image data	Relatively weak modeling of spatial locality and translational invariance

**Table 4:** Detailed architecture of proposed LcmUNet.

Stage	Layer	Type	Channel (In)	Channel (Out)
	1	LDA-A	3	16
	2	LDA-B	16	32
Encoder	3	LDA-B	32	128
	4	LMLP	128	160
	5	LMLP	160	256
	6	LMLP	256	160
	7	Bilinear Up (×2)	160	160
	8	LMLP	160	128
	9	Bilinear Up (×2)	128	128
	10	LDA-B	128	32
Decoder	11	Bilinear Up (×2)	32	32
	12	LDA-B	32	16
	13	Bilinear Up (×2)	16	16
	14	LDA-A	16	3
	15	Bilinear Up (×2)	3	3
	16	SoftMax	3	1

**Table 5:** Comparison of model complexity on the ISIC2018 dataset, where the bolded font indicates the optimal result.

Network	Inf. Time (in ms)	Params (in M)	GFLOPs	Iou (%)	F1 (%)
UNet	54.96	31.04	54.74	80.17	88.71
DeepLabv3+	12.27	5.81	6.61	82.07	89.70
Fcn8s	25.09	14.76	25.50	78.26	87.14
SegNet	50.38	34.97	45.13	79.93	87.55
AttUNet	79.03	34.88	66.63	80.92	89.20
UNext	**3.99**	**1.47**	0.57	82.00	89.87
ResUnet	113.63	14.76	97.16	77.86	87.27
LcmUNet (Ours)	9.27	1.49	**0.49**	**85.19**	**91.81**

**Table 6:** Performance of LcmUNet on the three datasets, where the bold font indicates the best results. Re stands for recall; Pr stands for precision.

Network	ISIC2018				Kvasir-SEG				BUSI			
	Iou	F1	Re	Pr	Iou	F1	Re	Pr	Iou	F1	Re	Pr
UNet	80.17	88.71	86.61	91.50	76.20	86.13	85.82	87.55	52.12	67.80	65.21	76.36
DeepLabv3+	82.07	89.70	89.01	91.61	79.15	88.04	88.40	88.57	58.72	73.16	72.00	78.44
Fcn8s	78.26	87.14	85.58	90.25	59.96	74.50	75.29	76.54	53.31	68.45	66.00	75.40
SegNet	79.93	87.55	90.56	88.34	79.33	88.20	86.55	90.62	58.85	73.37	72.21	75.99
AttUNet	80.92	89.20	88.48	90.45	78.48	88.55	87.01	89.13	56.30	71.34	67.84	**77.14**
UNext	82.00	89.87	88.98	91.29	77.57	86.99	87.67	88.05	60.74	75.35	77.05	75.46
ResUnet	77.86	87.27	86.30	88.34	67.10	79.93	78.55	83.02	44.23	60.79	57.15	73.25
LcmUNet (Ours)	**85.19**	**91.81**	**92.07**	**92.99**	**81.89**	**89.92**	**88.93**	**91.79**	**63.99**	**77.37**	**79.96**	76.69

**Table 7:** Ablation studies for different modular structures. Bolded font indicates optimal results.

Network	Iou (%)	F1 (%)	Inf. Time (in ms)	Params (in M)	GFLOPs
UNet	80.17	88.71	54.96	31.04	54.74
Unet + LDA-A	81.69	89.63	8.20	**0.51**	**0.36**
Unet + LDA-A + B	83.30	90.59	9.98	0.62	0.37
Unet + LDA-A + LMLP	84.69	91.52	**9.14**	1.46	0.45
LcmUNet	**85.19**	**91.81**	9.27	1.49	0.49

**Table 8:** Ablation study of the number of LDA-A channels. Bolded font indicates optimal results. Re stands for recall; Pr stands for precision.

Number of Channels	Iou (%)	Re (%)	Pr (%)	F1 (%)
0	**85.19**	**92.07**	**92.99**	**91.81**
0.25	83.61	91.20	91.07	90.81
0.5	84.75	91.25	91.76	91.29

**Table 9:** Ablation studies for feature fusion. Bolded font indicates optimal results. Re stands for recall; Pr stands for precision.

Settings	Iou (%)	Re (%)	Pr (%)	F1 (%)
0/0	81.55	90.57	90.57	89.50
0/1	82.27	91.34	90.16	89.90
1/0	83.00	91.19	90.42	91.19
1/1	**85.19**	**92.07**	**92.99**	**91.81**

## Data Availability

The code in this article cannot be published due to privacy and can be obtained from the corresponding author upon reasonable request.
